# Supplementary material for: Genomic mutation profile in progressive chronic lymphocytic leukemia patients prior to first-line chemoimmunotherapy with FCR and rituximab maintenance (REM)
Source: PLoS One. 2021 Sep 10;16(9):e0257353. doi: 10.1371/journal.pone.0257353 (PMC8432772; doi:10.1371/journal.pone.0257353)

S1 Fig. MRD association with progression-free survival (A) and overall survival (B).

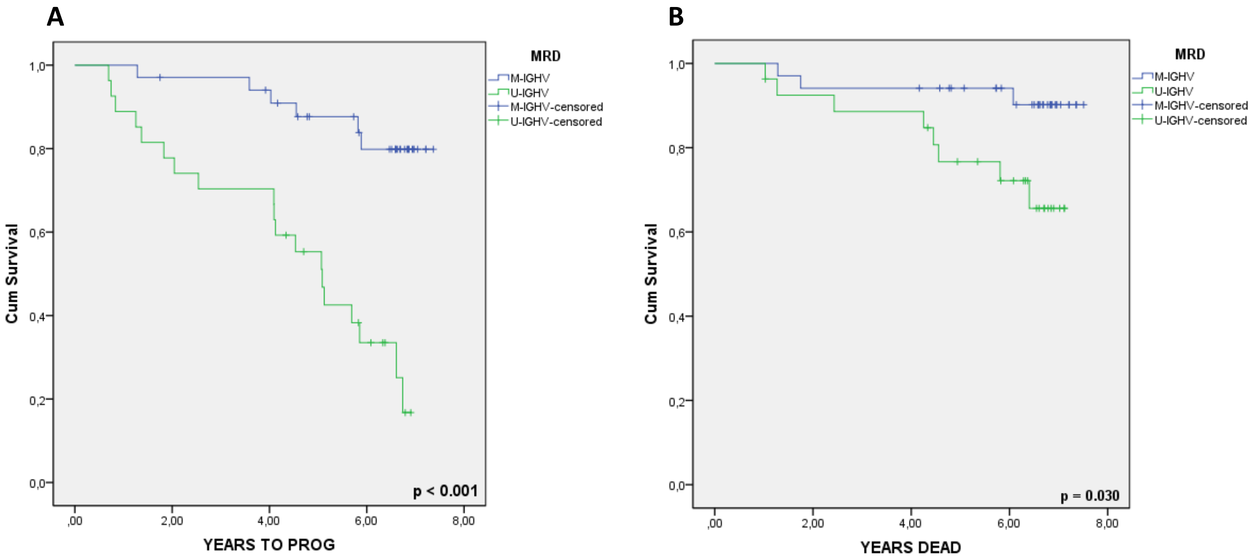

S2 Fig. Progression-free survival (PFS) and overall survival (OS). (A) PFS according to TP53/del(17p) and EGR2 status; **(B)** OS according to TP53/del(17p) status and Binet stage.

A

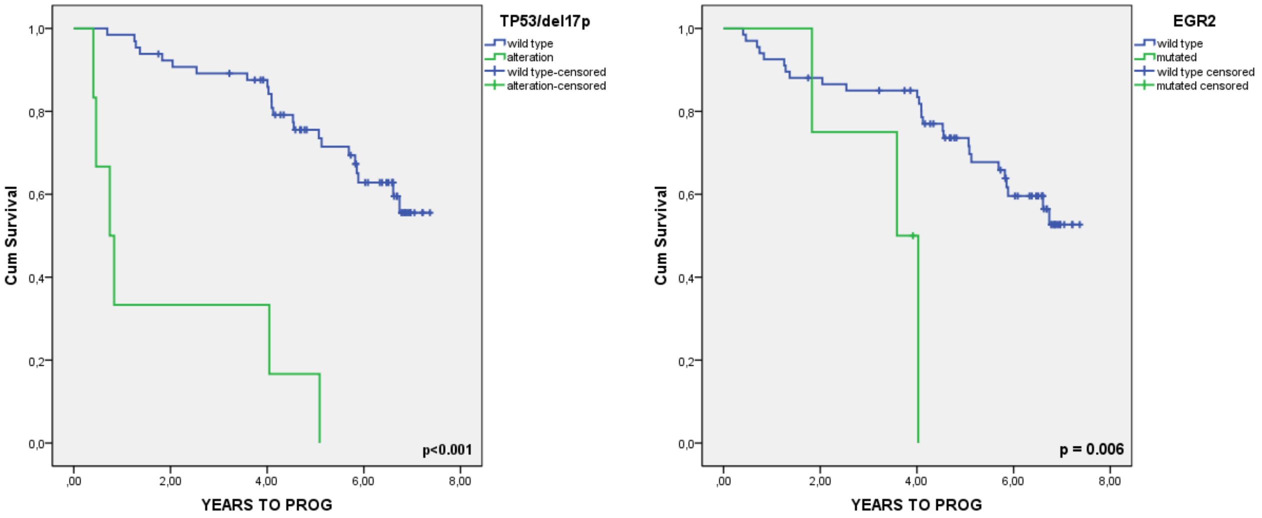

B

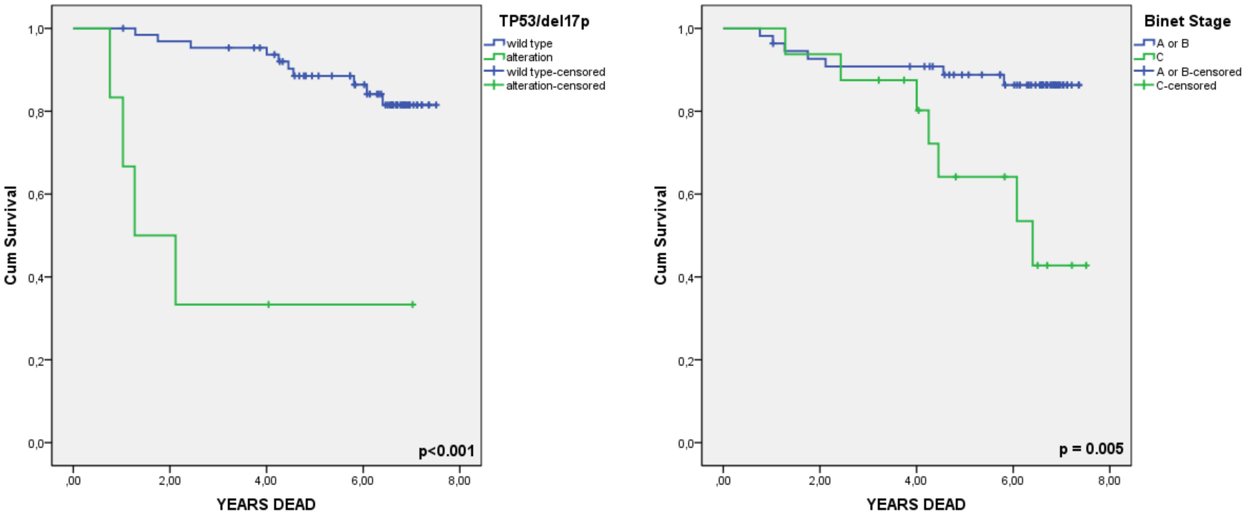

Supplement: S2 File — This PDF file contains S1 Fig: MRD association with progression-free survival (A) and overall survival (B). S2 Fig: Progression-free survival (PFS) and overall survival (OS). (PDF) [file pone.0257353.s002.pdf]
