# Supplementary material for: Genomic mutation profile in progressive chronic lymphocytic leukemia patients prior to first-line chemoimmunotherapy with FCR and rituximab maintenance (REM)
Source: PLoS One. 2021 Sep 10;16(9):e0257353. doi: 10.1371/journal.pone.0257353 (PMC8432772; doi:10.1371/journal.pone.0257353)
Supplement: Attachment — Submitted filename: Rebuttal letter_PONE-D-21-13505R1.docx [file pone.0257353.s004.docx]

Margarita Sánchez-Beato, PhD

IIS Puerta de Hierro-Segovia de Arana

e-mail: [msbeato@idiphim.org](mailto:msbeato@idiphim.org)

José A. García-Marco, MD, PhD

HU Puerta de Hierro-Majadahonda

e-mail: [jagarciam@aehh.org](mailto:jagarciam@aehh.org)

August 5, 2021

Dear Dr. F Bertoni,

As asked for by Reviewer 1, we have included a sentence indicating the variables included in the logistic regression analysis (page 14).

We greatly appreciate the revision and comments of the reviewers concerning our manuscript “*Genomic mutation profile in progressive chronic lymphocytic leukemia patients prior to first-line chemoimmunotherapy with FCR and rituximab maintenance (REM)”*.

Yours sincerely,

Margarita Sánchez-Beato, PhD

José A. García Marco, MD, PhD
